# Determination of Trophic Structure in Selected Freshwater Ecosystems by using Stable Isotope Analysis

**DOI:** 10.21315/tlsr2017.28.2.2

**Published:** 2017-07-31

**Authors:** ‘Amila Faqhira Zainordin, Suhaila Ab Hamid

**Affiliations:** School of Biological Sciences, Universiti Sains Malaysia, 11800 USM Pulau Pinang, Malaysia

**Keywords:** Stable Isotope Signature, Trophic Level, River, Rice Field

## Abstract

Stable isotope analysis has been used extensively to establish trophic relationships in many ecosystems. Present study utilised stable isotope signatures of carbon and nitrogen to identify trophic structure of aquatic food web in river and rice field ecosystems in Perak, northern peninsular Malaysia. The mean δ^13^C values of all producers ranged from −35.29 ± 0.21 to −26.00 ± 0.050‰. The greatest δ^15^N values noted was in zenarchopterid fish with 9.68 ± 0.020‰. The δ^15^N values of aquatic insects ranged between 2.59 ± 0.107 in Elmidae (Coleoptera) and 8.11 ± 0.022‰ in Nepidae (Hemiptera). Correspondingly, with all the δ^13^C and δ^15^N values recorded, it can be deduced that there are four trophic levels existed in the freshwater ecosystems which started with the producer (plants), followed by primary consumer (aquatic insects and non-predatory fish), secondary consumer (invertebrate predators) and lastly tertiary consumer (vertebrate predators).

## Abstract (ru)

Analisis isotop stabil telah digunakan secara meluas untuk mewujudkan hubungan trofik dalam pelbagai ekosistem. Kajian ini menggunakan tanda isotop stabil karbon dan nitrogen untuk mengenal pasti jaringan makanan akuatik dalam ekosistem sungai dan sawah padi di Perak, utara Semenanjung Malaysia, dan juga untuk menentukan struktur trofik jaringan makanan yang telah dikenal pasti. Nilai min δ^13^C untuk semua pengeluar berjulat dari −35.29 ± 0.21 hingga −26.00 ± 0.050‰. Nilai δ^15^N yang terbesar terdapat dalam ikan zenarchopterid dengan 9.68 ± 0.020‰. Nilai δ^15^N serangga akuatik berjulat antara 2.59 ± 0.107 dalam Elmidae (Coleoptera) dan 8.11 ± 0.022‰ dalam Nepidae (Hemiptera). Sehubungan dengan itu, dengan semua nilai δ^13^C dan δ^15^N yang telah direkod, ia boleh disimpulkan bahawa terdapat empat tahap trofik yang wujud dalam ekosistem air tawar yang bermula dengan pengeluar (tumbuh-tumbuhan), diikuti oleh konsumer primer (serangga akuatik dan ikan bukan pemangsa), konsumer sekunder (pemangsa invertebrata) dan akhir sekali konsumer tertier (pemangsa vertebrata).

## INTRODUCTION

Freshwater ecosystems include rivers, streams, lakes, freshwater swamps, peat swamps, rice fields and pools. As rivers flowing to low reaches, their water quality, substrates and food sources for aquatic organisms altered as well. Food web studies have been used to understand linkage in energy flow between aquatic ecosystems and terrestrial ecosystems and integrate organic matter processing (Hershey et al. 2010). Different food sources are consumed by different faunas due to their morphology, digestibility and the hydrology.

The stable isotope approach has become broadly used in ecology study, providing the possibility of obtaining objective and repeatable measures of trophic position, food chain and length omnivory (Cabana & Rasmussen 1994). The isotopic approach is based on isotopic concentration in the consumers’ tissues that resemble the isotopic composition in their diet (De Niro & Epstein 1978; 1981; Peterson & Fry 1987), which create of the relative contributions of isotopically different sources to the consumers’ diet (Fry 2006). Stable isotope of carbon (δ^13^C) is used to identify the ultimate source of carbon, or the primary energy source for a group of organisms or for an ecosystem (Fry & Sherr 1984), while nitrogen (δ^15^N) become enriched when transferred through a food web by means of feeding and predation (Peterson & Fry 1987).

The study on establishment of food web structure via stable isotope analysis is neglected in Malaysia mainly due to lack of proper facilities or instruments. Earlier findings on trophic structure was rather general, where plants were the producers and animals were the consumers that inhabit higher trophic level. However, this information lacks specific taxa of the organisms living in a particular habitat, especially in rivers and paddy fields, as different species of consumer might consume different type of food. Nevertheless, the application of stable isotope analysis was previously used to determine nutrition of prawns in mangroves (Newell *et al*. 1995), food preference of the giant mudskipper (Zulkifli *et al*., 2012) and food web of mudflats (Zulkifli *et al*. 2014). Recently, Dhiya Shafiqah (2014) attempted to establish the food web of aquatic insects in forested tropical streams. Therefore, this study aimed to identify the food web and to generally construct the trophic structure in the freshwater ecosystems by using stable isotope analysis.

## METHODOLOGY

### Study Sites

**Figure 1:**
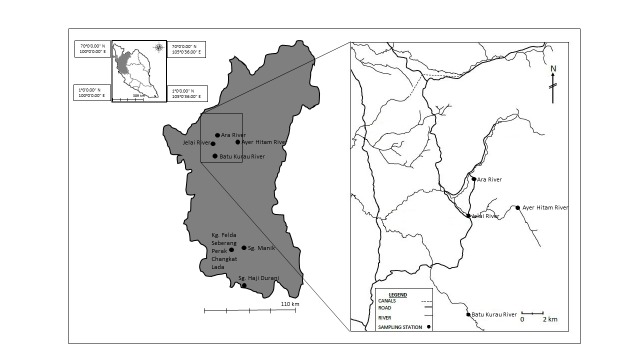
Map of study sites.

Samples for stable isotope analysis were collected from two different water bodies of rivers and rice fields (Figure 1). For rivers, samples were collected from four rivers in Bukit Merah, Perak, Malaysia: Batu Kurau River (04.54.17.400N, 100.49.59.900E), Ara River (05.05.25.500N, 100.51.10.700E), Jelai River (05.00.49.800N, 100.48.37.400E) and Ayer Hitam River (05.01.33.300N, 100.83.49.900E). For rice fields, samples were collected from three different rice fields in Perak, Malaysia with different paddy growth stages: rice fields of Sungai Haji Durani (tiller phase) (03.43.40N, 101.05.24E), Sungai Manik (post-harvest phase) (04.06.027N, 101.05.305E) and Kampung Felda Seberang Perak Changkat Lada (mature phase) (04.04.805N, 100.88.999E).

### Sample Collection and Preparation

Ten samples in each sampling site were collected for this study. Several dominant families in each study area were collected to represent each trophic level in their food web. Samples of plants that represents the producer; aquatic macroinvertebrates as the primary and secondary consumer; and fish as tertiary consumer were collected randomly in all study sites for stable isotope analysis to compare the trophic levels in the food web. Samples preparation for the analysis was adopted from methods described by Jardine *et al.* (2003) and Salas and Dudgeon (2001). All collected samples were cleaned, oven dried at 50°C–60°C for two days and the tissues were ground into fine, homogenous powder using a mortar and pestle. Ground samples were kept in small vials and stored in freezer until they were analysed. Samples in powdered form were sent to Doping Control Centre (DCC) in Universiti Sains Malaysia, analysed for stable carbon and nitrogen isotopes that was measured with an elemental analyser (EA), connected to an isotopic-ratio mass spectrometer (IR-MS). Stable isotope analysis followed a standard procedure by Carter and Barwick (2011). Urea isotopic working standard (C-13, N-15) was used as the standard, while USGS40 and USGS41 (carbon and nitrogen isotopes in L-glutamic acid) was used as isotopic reference material (RM). By following the manual advised by Coplen (2011), USGS40 was used to plot a calibration curve of stable carbon (δ^13^C) and nitrogen (δ^15^N). The curve was used to calculate the unknown carbon- and nitrogen- bearing substances measured with an elemental analyzer (EA) and an isotope-ratio mass spectrometer (IRMS) by quantifying drift with time and quantifying isotope-ratio-scale contraction when used together with USGS41 L-glutamic acid enriched in ^13^C and ^15^N. A pair of USGS40 and USGS41 RMs can be used at the beginning, the middle and the end of the analysis sequence to enable satisfactory scale correction and correction of drift with time (Coplen 2011). These reference materials and blanks should be interspersed in between 10–15 samples. Each samples were replicated and measured twice to obtain the mean for each data. Isotopic compositions of carbon and nitrogen were expressed in δ notation (δ^13^C and δ^15^N) as part per thousand (‰) differences from international standards – Vienna PeeDee Belemnite for carbon and atmospheric N_2_ for nitrogen. Stable isotope data were expressed as the relative difference between ratios of a sample and a standard using the equation:

$$\delta \times (\%)=\left[\left(\frac{\text{R sample}}{\text{R standard}}\right)\right]-1\times 1000$$

where X is δ^13^C or δ^15^N and R is ^13^C/^12^C or ^15^N/^14^N of sample or standard.

### Statistical Analysis

Homogeneity of variances and normality of the samples were checked in all instances. Since the data was normally distributed, to determine where significant differences lay between the samples in the rivers and paddy fields and between trophic level, one-way ANOVA for each sample were tested with Tukey post hoc tests, for both δ ^13^C and δ ^15^N.

## RESULTS

Stable isotope analysis was conducted on biological samples of plants, aquatic insects and fish available from the study areas. For each sample, the mean δ^15^N and δ^13^C values obtained displayed various degree of trophic position occurred in the rivers (Tables 1–2). The mean values of δ^15^N ranged between 2.59 ± 0.107‰ in Elmidae and 9.68 ± 0.020‰ in Zenarchopterid fish and for δ^13^C values ranged from − 33.08 ± 0.210‰ in Heptageniidae to − 15.03 ± 0.022‰ in Tipulidae.

**Figure 2:**
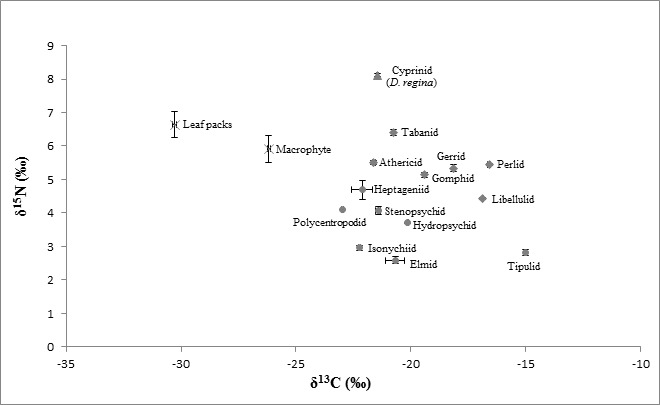

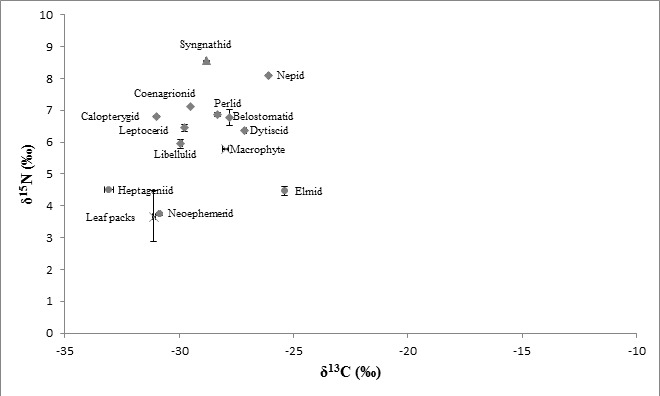

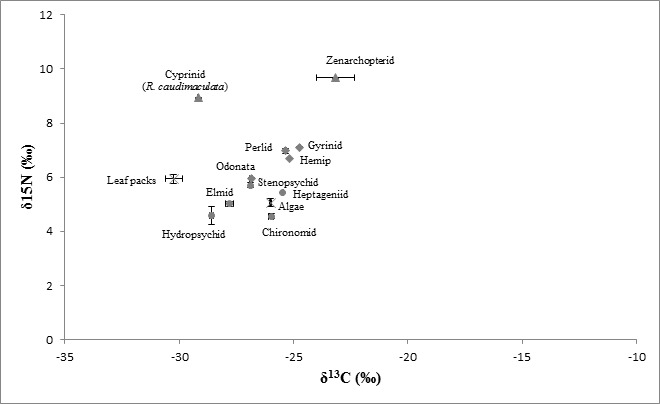

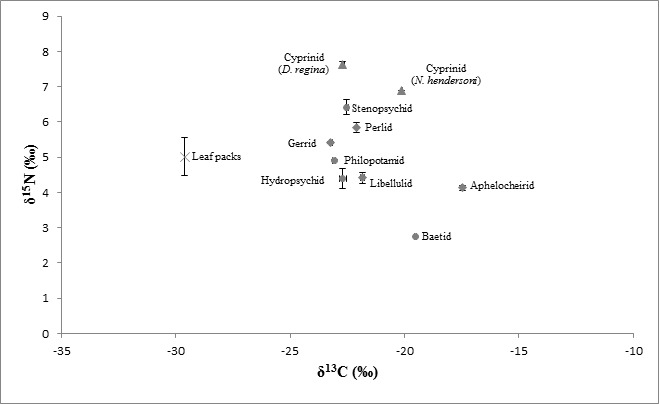
Dual isotopic plots of δ^15^N and δ^13^C of plants, aquatic insects and fish from rivers in Perak: 2 (a) Batu Kurau River, 2 (b) Jelai River, 2 (c) Ara River, 2 (d) Ayer Hitam River. Key of marker shapes: x – producers; circle – primary consumers; diamond – secondary consumers; triangle – tertiary consumers.

The δ^15^N values of vertebrate predators, i.e., fish recorded the greatest δ^15^N values among all consumers. As the values of δ^15^N increased with the increasing trophic levels, δ^15^N values of fish predators ranged between 7.63 ± 0.073 and 9.68 ± 0.020‰, which made them occupied the highest trophic level cum top predator and tertiary consumers in the aquatic food web (Figure 2). While the invertebrate predators, mostly the plecopterans, odonates and hemipterans scored δ^15^N values between 4.10 ± 0.010 and 8.11 ± 0.022‰, which made them lined below trophic level of fish, making them the secondary consumers in the food web. Following below them was the primary consumers, which were generally the herbivorous aquatic insects. They ranged from 2.59 ± 0.107 to 6.42 ± 0.214‰.

The δ^13^C signatures connote the significance of allochthonous and autochthonous sources of carbon. Assorted allochthonous leaf litters and autochthonous algae and aquatic macrophytes were expected to be the main basal food sources for the aquatic insects in the rivers. The average δ^13^C values for the leaf litters ranged from −31.11 ± 0.052 to −29.62 ± 0.012‰, while the autochthonous sources ranged between −26.00 ± 0.050 and −27.97 ± 0.125. So apparently, the amount of carbon in autochthonous food sources were greater than the allochthonous sources.

Dual isotopic plot of carbon and nitrogen in Figure 2 illustrates the energy flow and trophic structure of all organic samples available in the rivers. In this dual plot, the organic plants were expected to be the main local primary producers. By referring to Figure 2, the nitrogen and carbon signatures of all aquatic insects were clumped closely together, ranging from 2.59 ± 0.107 to 8.11 ± 0.022‰ for δ^15^N signatures and from −15.03 ± 0.022 to −33.08 ± 0.210 for δ^13^C signatures. Hence, according to these carbon and nitrogen values, it suggested that there are four major trophic levels in this river ecosystem that started with the primary producers, followed by the herbivorous aquatic insects, invertebrate predators and ended with vertebrate predators. In other terms, plants → aquatic insects → aquatic insect predators → fish predators.

Aquatic insects composition in the rice fields varied considerably from that of the rivers. The aquatic insects that inhabit this type of freshwater ecosystem only consisted of collector-gatherers and predators (Tables 3–4). The mean values of δ^15^N ranged from 3.58 ± 0.16 in algae to 10.72 ± 0.05‰ in osphronemid fish, while the values of δ^13^C ranged between −35.29 ± 0.21 in algae and −23.59 ± 0.07 in Nepidae.

The values of δ^15^N of osphronemid fish, *Parosphromenus deissneri* recorded the greatest δ^15^N values of 10.72 ± 0.05‰, which made them the top predators in the rice fields. The chain was followed by the aquatic insect predators, mainly the odonates and hemipterans, with δ^15^N values ranged between 2.58 ± 0.06 and 7.75 ± 0.00‰. Beneath this trophic level was the primary consumers, i.e., the collectors (Chironomidae) that consume suspended particulate organic matters in the rice fields. Algae were located at the base of the food web as the primary producer, which contained enriched carbon (−35.29 ± 0.21‰) that act as the energy source for the aquatic insects inhabiting rice field waters.

**Figure 3:**
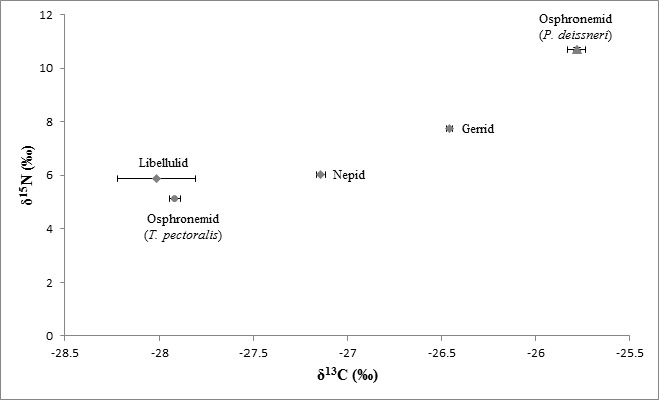

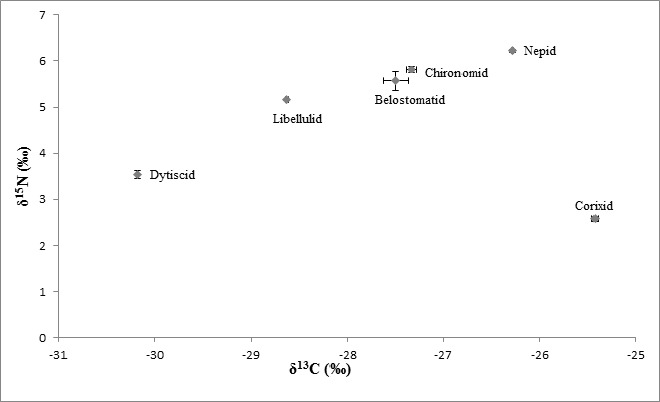

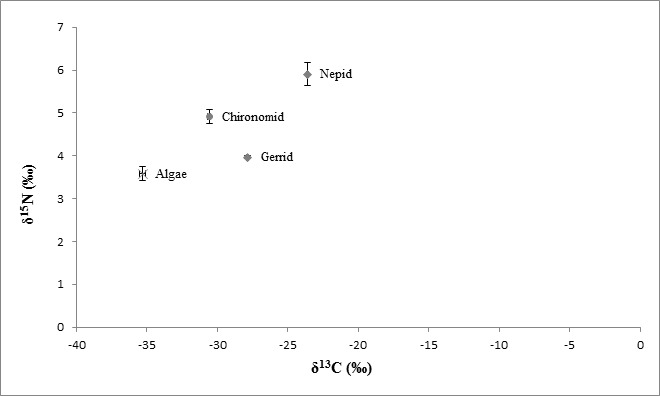
Dual isotopic plots of δ^15^N and δ^13^C of plants, aquatic insects and fish from rice fields in Perak: 3 (a) Sg. Haji Durani, 3 (b) Sg. Manik, 3 (c) Kg. Felda Seberang Perak Changkat Lada. Key of marker shapes: x – producers; circle – primary consumers; diamond – secondary consumers; triangle – tertiary consumers.

By referring to Figure 3, algae were positioned far at the base of the trophic structure and served as one of the main food sources. While the aquatic insects that clumped tightly on the dual isotopic plot acted as the primary (collectors) and secondary consumers (invertebrate predators), with δ^15^N ranged from 2.58 ± 0.06 to 7.75 ± 0.00‰ and δ^13^C ranged from −30.18 ± 0.01 to −23.59 ± 0.07‰. Nevertheless, the other fish species, *Trichopodus pectoralis*, had lower nitrogen values, as the same as the aquatic insects (5.12 ± 0.03‰) due to its herbivorous feeding mechanism. Therefore, based on the δ^15^N and δ^13^C values plotted, it was predicted that rice fields ecosystem also consisted of four major trophic levels, similar to river ecosystems, yet with simpler and less intricate food web, specifically, producers → aquatic insects (and non-predatory fish) → aquatic insect predators → fish predators.

There was a statistically significant difference between samples in all study sites as determined by one-way ANOVA (*F*_(6, 62)_ = 2.69, *P* = 0.022) for δ^15^N and (*F*_(6, 62)_ = 15.35, *P* = 0.000) for δ^13^C. Tukey post hoc test performed on one-way ANOVA for each sample established, where the values of δ^15^N and δ^13^C were differed between sites and trophic levels.

## DISCUSSION

Analysing the stomach contents reveal the taxa of prey they consume during the right time preceding capture of animals (Munoz-Gil *et al*. 2013). However, in this method the movements of nutrients and matter through food webs and ecosystems are often difficult to observe or quantify (Polis 1991). Alternatively, stable isotope analysis is used to elucidate trophic relationships. Carbon and nitrogen stable isotopes are useful to trace energy sources and food web structure in ecosystems. It also shows the effects of anthropogenic stress on aquatic ecosystems (Bergfur *et al*. 2009). The stable isotope approach is based on the similarity of isotopic concentration in the consumers’ tissues to the stable isotopic composition of their diet (De Niro & Epstein 1978; 1981; Peterson & Fry 1987). Accordingly, it establishes the relative contributions of isotopically different sources to the diet of consumers (Fry 2006).

The contents of both isotopes in organisms varied inconsiderably in different rivers. This might due to the impact from the nearby land uses towards the river and different basal resources they consumed. Changes in δ^15^N could specify changes in nutrient delivery to aquatic ecosystems (Cole *et al*. 2004). δ^15^N signatures of aquatic macrophyte and algae were higher than that of the insects suggested autochthonous origin (Salas & Dudgeon 2001). Decreasing in stream discharge during dry season would increase the concentrations of phosphate and nitrate in the river (Dudgeon 1984, 1992; Dudgeon & Corlett 1994), and hence might clarify high concentration of nitrogen in the producers although none of the seasons was included in this study. Moreover, Thomas and Daldorph (1994) stated that high nutrient availability is known to enhance primary production of filamentous algae and periphyton. Next, Heptageniidae (non-predatory, herbivorous scrapers) in Ara River contained the highest δ^15^N value of 5.45 ± 0.006‰ (4.69 ± 0.279‰ in Batu Kurau River; 4.50 ± 0.004‰ in Jelai River). During the sample collections, Ara River had numerous algae grown on the stony substrates that act as the potential food source for the heptageniids. According to Salas and Dudgeon (2001), high nitrogen content in the heptageniids might probably because of the abundance of autochthonous algae grown on the stone surfaces that could provide more energy for the insects. Such ^15^N-enrichment of autochthonous sources can be explained by nitrogen inputs from surrounding orchard (Macko & Ostrom 1994) in the human settlements. Indeed, as the nitrogen content increased with the increasing trophic levels, the predatory Cyprinidae’s δ^15^N content in present study was rather similar to the values reported for cyprinids in other study. For example, Dhiya Shafiqah (2014) found that the cyprinids in undisturbed rivers in Royal Belum State Park scored δ^15^N values of 8.45 ± 0.177‰.

In freshwater ecosystems, the δ^13^C is often used to distinguish or trace the relative importance of allochthonous and autochthonous sources of carbon (Rounick & Winterbourn 1986). It only shows little variation among trophic levels (Fry 2006). Leaf litters, algae and aquatic macrophytes were expected to be the main source of organic carbon and nutrients for the aquatic organisms inhabiting the rivers. Among all sites sampled, Batu Kurau and Ayer Hitam rivers had more leaf litters as compared to Jelai and Ara River (which located at higher order stream) that provide allochthonous sources to the rivers. This can be likely linked to the shaded canopy cover in Batu Kurau and Ayer Hitam rivers. The increased allochthonous carbon input from the surrounding riparian vegetation in Batu Kurau and Ayer Hitam rivers would act as major source of organic carbon and nutrients for organisms within these sites and which is lacking at higher order stream reach. Leaf litters were also source of coarse and fine particulate organic matters for the collectors as they did not consume the plants directly. Previous studies by England and Rosemond (2004) and Kominoski *et al.* (2011) discovered that the composition of leaf litters influenced the structure and function of stream ecosystem by altering the nutrient content and energy transfer in the food web in forest stream ecosystem. Present study showed that leaf litters contained lower carbon signatures than the algae and aquatic macrophytes did, as autochthonous food source had more enriched carbon than allochthonous sources (Salas & Dudgeon 2001). Previous studies also observed similar carbon enrichment of autochthonous sources, as reported by Bunn *et al.* (1999), Dhiya Shafiqah (2014), Lester *et al.* (1995) and Thorp *et al.* (1998).

Gregory *et al*. (1987) found the reduction of canopy cover had exposed stream surface to direct sunlight and thus influenced the aquatic invertebrates to use more autotrophic energy sources. In this study, Jelai and Ara rivers and also rice fields had open canopy covers. The increasing growth of aquatic vegetation had interrupted the aquatic invertebrate abundance and species richness by altering their functional role in the ecosystem, becoming the consumer of organic material and served as preys to larger organisms (Collier 2002; Nelson & Lieberman 2002; Quinn *et al*. 1997; Suren *et al*. 2003). Hence, supplementary to the allochthonous sources, as a primary producer was also represented by other two types of autochthonous sources: aquatic macrophyte (available in Batu Kurau and Jelai rivers) and periphytic algae (which grown abundantly in Ara River). Autochthonous foods had higher quality (with lower C/N ratios and higher essential fatty acids contents) than leaf litter (Lau *et al*. 2008, 2009) which probably accounted for their importance to consumers. In this study, the autochthonous carbon had more ^13^C-enriched than allochthonous sources. Algal foods also have a tendency to be the main energy source of stream consumers in the Neotropics (Brito *et al*. 2006; Bunn *et al*. 1999; March & Pringle 2003) and even in some temperate lotic ecosystems (Bunn *et al*. 2003; Delong & Thorp 2006; Torres-Ruiz *et al*. 2007).

Higher nutrient availability could enhance primary production of filamentous algae and periphyton (Thomas & Daldorph, 1994) thus increasing the assimilation of dissolved inorganic carbon (CO_2_). Likewise, algae were reported to be the important primary producer in autochthonous pathway that supplied energy sources to aquatic insects (Newell *et al*. 1995). Consequently, ^13^C-enrichment of aquatic macrophyte and algae in Batu Kurau, Jelai and Ara rivers reflect the combined effect of light and nutrients on their growth in the environment.

In contrast to the river ecosystem, only algae were found to be the basal food sources for the aquatic insects in rice field. Brito *et al*. (2006), Bunn *et al.* (1999) and March and Pringle (2003) stated that algae have a tendency to be the major energy source for aquatic consumers in the Neotropics and temperate ecosystems. The rice field offered a wide variety of conditions for the growth of algae. Several factors including high temperature, nutrient availability, conditions of soil, humidity and the ability of the algae to withstand desiccation (Roger & Reynaud 1979) favour the growth of algae in rice fields. According to Singh (1961) and Venkataraman (1972), the growth of algae contributed significantly to spontaneous fertility of paddy soils. Fogg *et al.* (1973) stated that since algae are capable of both photosynthesis and nitrogen fixation in aerobic conditions, such trophic independence regarding carbon and nitrogen, combined with a great adaptability to variations in edaphic factors, permits algae to be omnipresent and at the same time gives them a unique potential to contribute productivity in a variety of agricultural and ecological situations. In this study, plants (algae in Changkat Lada rice field) was trophically located as basal source with mean value of δ^15^N ~ 3.58 ± 0.16‰. Algae contained the most ^13^C-enriched food source with −35.29 ± 0.21‰ thus make it an essential primary producer in autochthonous pathway that provide energy source to the aquatic invertebrates (Newell *et al*. 1995).

The use of δ^15^N as organisms’ trophic position tracer and organic source information (Peterson 1999) had shed light upon many difficulties in estimating trophic position (Vander Zanden *et al*. 1997). δ^15^N signifies the major energy flow pathways that offer a time-integrated measure of trophic positions, account for spatial and temporal variations in feeding at multiple levels in food web and detect trophic interactions that are otherwise would be unnoticeable (Vander Zanden *et al.* 1997).

Aquatic insect family richness was greater in Batu Kurau River, later found to have the greatest canopy cover (60%). This suggested that increased canopy cover is related to creating more complex habitat for a wider variety for macroinvertebrates (VanDongen *et al*. 2011). Increased richness could be due to higher amounts of allochthonous input from the terrestrial landscape, which would also account for greater representation of the shredders, collector-gatherers and collector-filterers. The higher abundance of leaf litter in Batu Kurau and Ayer Hitam rivers created a larger energy source for the collector-gatherers and collector-filterers, which were represented in large number of Hydropsychidae and Stenopsychidae families. Less open areas supported the scrapers (Vannote *et al*., 1980) in Batu Kurau River. The aquatic insects ranged widely in their δ^13^C values, and typically were located trophically in the different level above the producers as primary and secondary consumers, but below the tertiary consumers (vertebrate predators). The wide δ^13^C range of aquatic macroinvertebrates indicated there were multiple food sources (plants or cannibalism) in the aquatic environment (the aquatic insects carbon signatures ranged widely from −15.03 to −22.98 in Batu Kurau River; −25.40 to −33.08 in Jelai River; −17.49 to −23.22 in Ayer Hitam River). In Ara River, the carbon signatures of aquatic insects did not vary widely upon consumers (−24.73 to −28.59 δ^13^C). This small range of δ^13^C values suggested a distinct utilisation of carbon sources for each individual.

Generally, the collector-gatherer of Elmidae (in Batu Kurau River), Neoephemeridae (in Jelai River), Chironomidae (in Ara River) and Baetidae (in Ayer Hitam River) were located at the lowest trophic level among all aquatic insects group. Then, followed by other aquatic insect families comprised of different functional guilds (collector-filterer, shredder, scraper and predator). They were clumped closely together in the dual plot because they are positioned trophically in the same level and their large range of δ^13^C values implies different consumption of carbon sources for each individual. Furthermore, unlike *P. desissneri*, *T. pectoralis* had almost the same nitrogen value as the collector-gatherers (chironomid), which ranged between 4.91 to 5.81‰. This proved that *T. pectoralis* did not prey on other aquatic insects; in fact, they consume mostly plant matter and algae (Ambak *et al*. 2010). They are adaptable species that can survive in a wide range of biotopes; however, they tend to thrive best in slow-moving or still waters where submerged vegetation grows densely, for example, in rice fields, swamps and irrigation canals (Ambak *et al*. 2010).

In the rice fields, collector-gatherers, particularly chironomid larvae, were the common primary consumer found in the study areas (Sg. Manik and Changkat Lada). Chironomidae are common insects during the wet phase of the rice growing season (Al-Shami *et al*. 2008) and reduced in abundance towards tiller and pre-harvest phase (Che Salmah & Abu Hassan 2002). In this study, the chironomid larvae present in paddy fields of Sg. Manik (post-harvest phase) and Changkat Lada (tiller) were quite low in abundance. This functional guild generally located at the lowest trophic level among all aquatic insects group, with δ^15^N ~ 5.81 ± 0.06‰ and 4.91 ± 0.17‰ in Sg. Manik and Changkat Lada, respectively; just above the algae with mean value of δ^15^N ~ 3.58 ± 0.16‰.

Then, these primary consumers were preyed by the secondary consumers, which were the invertebrate predators, mostly of plecopterans, odonates and hemipterans. Their nitrogen signatures proved that they are located one trophic level above other aquatic insects, yet underneath the tertiary consumers, or the underwater top predators: the fish. Odonates tend to consume different types of insect to reduce prey overlapping among genera (Motta & Uieda 2004). Moreover, Odonata are also able to ingest various kinds of prey from different size classes (Dudgeon 1995). On the other hand, in this lentic ecosystem, there were only two functional feeding groups available, specifically collector-gatherer and predator; in which differed from lotic ecosystem. During post-harvest phase in Sg. Manik, the abundance of aquatic insect larvae was greatly reduced after the paddy plants were harvested and the field became almost dry. During this stage, Corixidae (Hemiptera) was found in large abundance in this rice field with 46.67% of the total individuals collected. They inhabit standing water and were omnivores feeding on algae, detritus and chironomid larvae (Yule & Yong 2004). Due to this feeding habit, they recorded the least δ^15^N value with 2.58 ± 0.06‰, which was lesser nitrogen signature than their prey: the chironomids. Dytiscids found in Sg. Manik rice field scored mean δ^15^N value of 3.54 ± 0.09‰ which also had lower nitrogen signature than the collector-gatherer. Even though they were carnivores, dytiscid beetles sometimes are scavengers too depending on the availability of the prey (Yule & Yong, 2004).

Most hemipterans and libellulids (Odonata) were the aquatic insect predators based on their morphology and behaviour. The hemipteran families: Nepidae, Belostomatidae and Gerridae are efficient hunters and have been known to prey on aquatic insect larvae, small fish and tadpoles (Morse *et al*., 1994). They were presented at all study sites during both phases of paddy and they prefer calm, standing water of rice fields; especially the belostomatid bugs (Yule & Yong 2004). Libellulids (Odonata) occurred in both paddy phases: tiller and post-harvest. They inhabit not only fast streams and rivers but also wide range of still or sluggish waters; including pools, lakes and ponds. Their larvae were tolerant to wide fluctuations in surrounding environment conditions for example, temperature, oxygenation and pH (Yule & Yong 2004). The larvae of most libellulid species are voracious carnivores and are secretive, hiding among vegetation at the bottom (Gillott 2005), using their vision and/or mechanoreceptors to detect (Yule & Yong 2004) and ambush their prey. Naturally, the prehensile labium is shot out very rapidly to capture the target organisms. Libellulids from genus *Orthetrum* (found abundant in rice field of Sg. Haji Durani) feed on other odonates species (cannibalism) and sometimes even larger than themselves (Yule & Yong 2004).

Concomitantly, fish families of Cyprinidae, Syngnathidae, Zenarchopteridae and Osphronemidae were the top underwater predator found in the study areas. The cyprinids prefer clear, shallow water with a sandy bottom. Batu Kurau, Ara and Ayer Hitam rivers provided excellent habitat for this family because the water was clear and there were only few areas with cobble substrate. Three species of cyprinids were sampled for this study: *Devario regina* (Queen danio), *Rasbora caudimaculata* (Greater scissortail) and *Neolissochilus hendersoni* (Copper Mahseer). The syngnathids: *Doryichthys deokhatoides* (Freshwater pipefish) however, preferred slow water current, grasses, roots or shore vegetation. Jelai River provided an ideal habitat for the syngnathids. The zenarchopterids: *Zenarchopterus sp.* (Freshwater halfbeak) preferred a shallow water column and would typically orient them into the water current and consume aquatic insect larvae and small insects that have fallen onto the water surface and these situations can be seen in Ara River. The Osphronemid: *Parosphromenus deissneri* (Deissner’s Liquorice Gourami) has been collected in Sg. Hj. Durani paddy field containing shallow, stagnant and muddy water. This species is chiefly a micropredator that feeds on aquatic invertebrates. As fish was assumed to be the top consumer in the river ecosystem, the isotopic model suggested by Post (2002) was used to calculate the estimation of trophic level of consumers.

According to Fry (1988) and Peterson and Fry (1987), the distribution of nitrogen signatures was proved to be an indicator of trophic structure as the δ^15^N increases consistently with the increasing trophic level of consumers. In his study, Fry (1988) stated that in a food web, ^15^N increases much more regular, with fish generally having higher values than invertebrates and piscivorous fish having the highest δ^15^N values. This suggested that ^15^N is more reliable trophic indicator than ^13^C. Minagawa and Wada (1984) had proposed three assumptions in order to estimate the trophic position: 1) for every increasing trophic level, the trophic fractionation of δ^15^N is 3.4‰; 2) the trophic fractionation of δ^13^C is near 0‰ and 3) carbon and nitrogen move through the food web with a similar stoichiometry. Thence by following the assumptions, generally four trophic levels were determined in both water bodies: the producer (the plants), primary consumer (herbivorous aquatic insects and non-predatory fish), secondary consumer (predatory aquatic insects) and tertiary consumer (predatory fish). Similar findings have been reported in recent studies by Dhiya Shafiqah (2014) from Malaysia and VanDongen *et al*. (2011) from the US.

## CONCLUSION

To conclude, the aquatic food web in freshwater ecosystems do have similar trophic structure in which, there are four major trophic levels identified in two different water bodies in this study. The “plants → aquatic insects (and non-predatory fish) → invertebrate predators → vertebrate predators” pathways apply to both river and rice field ecosystems. In fact, rivers had more complex food web as the aquatic inhabitants in the rivers were more diverse, compared to the aquatic faunas in the rice fields. Basal food sources were more abundant too in the river and thus making the food web in the river ecosystems were more intricate. By studying specific taxa and trophic levels in the ecosystem and once the specific targeted species is identified, any further studies and conservation efforts of the top predators can be done effectively. Future works should widen the range and level of detail of sampling at different times and study sites in order to include other potential food sources for the aquatic inhabitants.

## Figures and Tables

**Table 1:** Stable isotope ratios of δ^15^N in ‰ (mean ± se) from biological samples in selected rivers in Bukit Merah.

	Samples	Rivers			

		Batu Kurau	Jelai	Ara	Ayer Hitam
Plants	Algae	-	-	5.05 ± 0.144	-
	Aquatic macrophyte	5.92 ± 0.398	5.79 ± 0.007	-	-
	Leaf litters	6.64 ± 0.378	3.68 ± 0.792	5.94 ± 0.165	5.02 ± 0.530
Insects	Heptageniidae	4.69 ± 0.279	4.50 ± 0.004	5.45 ± 0.006	-
	Tipulidae	2.83 ± 0.091	-	-	-
	Baetidae	-	-	-	2.75 ± 0.006
	Chironomidae	-	-	4.56 ± 0.081	-
	Elmidae	2.59 ± 0.107	4.47 ± 0.145	5.01 ± 0.082	-
	Hydropsychidae	3.71 ± 0.015	-	4.59 ± 0.340	4.41 ± 0.281
	Isonychiidae	2.96 ± 0.075	-	-	-
	Neoephemeridae	-	3.75 ± 0.055	-	-
	Philopotamidae	-	-	-	4.92 ± 0.014
	Stenopsychidae	4.07 ± 0.111	-	5.71 ± 0.098	6.42 ± 0.214
	Aphelocheiridae	-	-	-	4.13 ± 0.064
	Athericidae	5.52 ± 0.060	-	-	-
	Belostomatidae	-	6.78 ± 0.254	-	-
	Calopterygidae	-	6.81 ± 0.032	-	-
	Coenagrionidae	-	7.11 ± 0.004	-	-
	Dytiscidae	-	6.37 ± 0.024	-	-
	Gerridae	5.33 ± 0.111	-	6.68 ± 0.094	5.41 ± 0.028
	Gomphidae	5.14 ± 0.072	-	-	-
	Gyrinidae	-	-	7.11 ± 0.023	-
	Leptoceridae	-	6.45 ± 0.112	-	-
	Libellulidae	4.42 ± 0.024	5.95 ± 0.137	5.96 ± 0.009	4.41 ± 0.15
	Nepidae	-	8.11 ± 0.022	-	-
	Perlidae	5.44 ± 0.033	6.86 ± 0.062	6.98 ± 0.091	5.85 ± 0.146
	Polycentropodidae	4.10 ± 0.010	-	-	-
	Tabanidae	6.40 ± 0.098	-	-	-
Fish	Cyprinidae (*Devario regina*)	8.11 ± 0.043	-	8.93 ± 0.010	7.63 ± 0.073
	Syngnathidae	-	8.55 ± 0.021	-	-
	Zenarchopteridae	-	-	9.68 ± 0.020	-

- = not available

**Table 2:** Stable isotope ratios of δ^13^C in ‰ (mean ± se) from biological samples in selected rivers in Bukit Merah.

	Samples	Rivers			

		Batu Kurau	Jelai	Ara	Ayer Hitam
Plants	Algae	**-**	**-**	−26.00 ± 0.050	**-**
	Aquatic macrophyte	−26.18 ± 0.078	− 27.97 ± 0.125	-	-
	Leaf litters	−30.29 ± 0.074	− 31.11 ± 0.052	− 30.24 ± 0.374	− 29.62 ± 0.012
Insects	Heptageniidae	−22.13 ± 0.463	− 33.08 ± 0.210	− 25.48 ± 0.021	-
	Tipulidae	−15.03 ± 0.022	-	-	-
	Baetidae	-	-	-	−19.53 ± 0.040
	Chironomidae	-	-	−25.97 ± 0.109	-
	Elmidae	−20.68 ± 0.410	− 25.40 ± 0.002	− 27.81 ± 0.167	-
	Hydropsychidae	−20.14 ± 0.025	-	− 28.59 ± 0.072	− 22.69 ± 0.150
	Isonychiidae	−22.24 ± 0.023	-	-	-
	Neoephemeridae	-	−30.87 ± 0.030	-	-
	Philopotamidae	-	-	-	−23.08 ± 0.054
	Stenopsychidae	−21.43 ± 0.110	-	−26.89 ± 0.034	−22.52 ± 0.080
	Aphelocheiridae	-	-	-	−17.49 ± 0.033
	Athericidae	−21.61 ± 0.049	-	-	-
	Belostomatidae	-	−27.82 ± 0.008	-	-
	Calopterygidae	-	−30.99 ± 0.017	-	-
	Coenagrionidae	-	−29.52 ± 0.005	-	-
	Dytiscidae	-	−27.13 ± 0.070	-	-
	Gerridae	−18.15 ± 0.019	-	−25.18 ± 0.001	−23.22 ± 0.066
	Gomphidae	−19.40 ± 0.003	-	-	-
	Gyrinidae	-	-	−24.73 ± 0.023	-
	Leptoceridae	-	−29.77 ± 0.031	-	-
	Libellulidae	−16.87 ± 0.026	−29.93 ± 0.065	−26.86 ± 0.078	−21.85 ± 0.095
	Nepidae	-	−26.10 ± 0.052	-	-
	Perlidae	−16.60 ± 0.051	−28.33 ± 0.003	−25.36 ± 0.049	−22.09 ± 0.027
	Polycentropodidae	−22.98 ± 0.042	-	-	-
	Tabanidae	−20.75 ± 0.021	-	-	-
Fish	Cyprinidae (*Devario regina*)	−21.44 ± 0.036	-	−29.17 ± 0.010	−22.71 ± 0.064
	Syngnathidae	-	−28.83 ± 0.015	-	-
	Zenarchopteridae	-	-	−23.10 ± 0.828	-

- = not available

**Table 3:** Stable isotope ratios of δ^15^N in ‰ (mean ± se) from biological samples in rice fields in Perak.

	Samples	Paddy fields		

		Sg. Hj. Durani	Sg. Manik	Kg. Felda Seberang Perak Changkat Lada
Plant	Algae	-	-	3.58 ± 0.16
Insects	Chironomidae	-	5.81 ± 0.06	4.91 ± 0.17
	Belostomatidae	-	5.57 ± 0.20	-
	Corixidae	-	2.58 ± 0.06	-
	Dytiscidae	-	3.54 ± 0.09	-
	Gerridae	7.75 ± 0.00	-	3.97 ± 0.03
	Libellulidae	5.87 ± 0.02	5.16 ± 0.01	-
	Nepidae	6.02 ± 0.00	6.22 ± 0.03	5.91 ± 0.26
Fish	Osphronemidae (*Trichopodus pectoralis*)	5.12 ± 0.03	-	-
	Osphronemidae (*Parosphromenus deissneri*)	10.72 ± 0.05	-	-

- = not available

**Table 4:** Stable isotope ratios of δ^13^C in ‰ (mean ± se) from biological samples in rice fields in Perak.

	Samples	Paddy field		

		Sg. Hj. Durani	Sg. Manik	Kg. Felda Seberang Perak Changkat Lada
Plant	Algae	-	-	−35.29 ± 0.21
Insects	Chironomidae	-	− 27.33 ± 0.05	−30.58 ± 0.10
	Belostomatidae	-	−27.49 ± 0.13	-
	Corixidae	-	−25.42 ± 0.03	-
	Dytiscidae	-	−30.18 ± 0.01	-
	Gerridae	−26.46 ± 0.02	-	−27.87 ± 0.06
	Libellulidae	−28.02 ± 0.21	−28.62 ± 0.01	-
	Nepidae	−27.14 ± 0.02	−26.28 ± 0.01	−23.59 ± 0.07
Fish	Osphronemidae (*Trichopodus pectoralis*)	−27.92 ± 0.03	-	-
	Osphronemidae (*Parosphromenus deissneri*)	−25.78 ± 0.05	-	-

- = not available

## References

[b1-tlsr-28-2-9] Ambak MA, Isa MM, Zakaria MZ, Ghaffar MA (2010). Fishes of Malaysia.

[b2-tlsr-28-2-9] Al-Shami SA, Che Salmah MR, Siti Azizah MN, Abu Hassan A (2008). Distribution and abundance of larval Chironomidae (Diptera) in a rice agroecosystem in Penang, Malaysia. Boletim do Museu Municipal do Funchal.

[b3-tlsr-28-2-9] Bergfur J, Johnson RK, Sandin L, Geodkoop W (2009). Effects of nutrient enrichmen on C and N stable isotope ratios of invertebrates, fish and their food resources in boreal streams. Hydrobiologia.

[b4-tlsr-28-2-9] Brito EF, Moulton TP, Souza ML, Bunn SE (2006). Stable isotope analysis in microalgae as the predominant food source of fauna in a coastal forest stream, south-east Brazil. Austral Ecology.

[b5-tlsr-28-2-9] Bunn SE, Davies PM, Mosisch TD (1999). Ecosystem measures of river health and their response to riparian and catchment degradation. Freshwater Biology.

[b6-tlsr-28-2-9] Bunn SE, Davies PM, Winning M (2003). Sources of organic carbon supporting the food web of and arid zone floodplain river. Freshwater Biology.

[b7-tlsr-28-2-9] Cabana G, Rasmussen JB (1994). Modelling food chain structure and contaminant bioaccumulation using stable nitrogen isotopes. Nature (Lond).

[b8-tlsr-28-2-9] Carter JF, Barwick VJ (2011). Good practice guide for isotope ratio mass spectrometry.

[b9-tlsr-28-2-9] Che Salmah MR, Abu Hassan A (2002). Distribution of aquatic insects in relation to rice cultivation phases in a rain fed rice field. Jurnal Biosains.

[b10-tlsr-28-2-9] Cole ML, Valiela I, Kroeger KD, Tomasky GL, Cebrian J, Wigand C, McKinney RA, Grady SP, da Silva MHC (2004). Assessment of a delta N-15 isotopic method to indicate anthropogenic eutrophication in aquatic ecosystems. Journal of Environmental Quality.

[b11-tlsr-28-2-9] Collier KJ (2002). Effects of flow regulation and sediment flushing on instream habitat and benthic invertebrates in a New Zealand river influenced by a volcanic eruption. River Research and Applications.

[b12-tlsr-28-2-9] Coplen TB (2011). Report of stable isotopic composition.

[b13-tlsr-28-2-9] Delong MD, Thorp JH (2006). Significance of instream autotrophs in trophic dynamics of the Upper Mississippi River. Oecologia.

[b14-tlsr-28-2-9] De Niro MJ, Epstein S (1978). Influence of diet on the distribution of carbon isotopes in animals. Geochimica Cosmochimica Acta.

[b15-tlsr-28-2-9] De Niro MJ, Epstein S (1981). Influence of diet on the distribution of nitrogen isotopes in animals. Geochimica Cosmochimica Acta.

[b16-tlsr-28-2-9] Dhiya Shafiqah R (2014). Habitat characterization, trophic position and seasonal influence on aquatic insects in the selected feeder streams of Belum–Temengor Forest Complex (BTFC), Perak. Unpublished MSc thesis.

[b17-tlsr-28-2-9] Dudgeon D (1984). Seasonal and long-term changes in the hydrobiology of the Lam Tsuen River, New Territories, Hong Kong, with special reference to benthic macroinvertebrate distribution and abundance. Archiv fur Hydrobiologie.

[b18-tlsr-28-2-9] Dudgeon D (1992). Patterns and processes in stream ecology.

[b19-tlsr-28-2-9] Dudgeon D, Cushing CE, Cummins KW, Minshall GW (1995). The ecology of rivers and streams in tropical Asia. River and stream ecosystems.

[b20-tlsr-28-2-9] Dudgeon D, Corlett R (1994). Hill and streams: An ecology of Hong Kong.

[b21-tlsr-28-2-9] England LE, Rosemond AD (2004). Small reductions in forest cover weaken terrestrial–aquatic linkages in headwater streams. Freshwater Biology.

[b22-tlsr-28-2-9] Fogg GE, Steward WDP, Fay P, Walsby AE (1973). The blue-green algae.

[b23-tlsr-28-2-9] Fry B (1988). Food web structure on Georges Bank from stable C, N and S isotopic compositions. Limnology and Oceanography.

[b24-tlsr-28-2-9] Fry B (2006). Stable isotope ecology.

[b25-tlsr-28-2-9] Fry B, Sherr EB (1984). δ^13^C measurements as indicators of carbon flow in marine and frashwater ecosystems. Contributions in Marine Science.

[b26-tlsr-28-2-9] Gillott C (2005). Entomology.

[b27-tlsr-28-2-9] Gregory SV, Lamberti GA, Erman DC, Koski KV, Murphy ML, Sedell JR (1987). Influence of forest practices on aquatic production.

[b28-tlsr-28-2-9] Hershey AE, Lamberti GA, Chaloner DT, Northington RM, Hershey AE, Lamberti GA, Chaloner DT, Northington RM (2010). Aquatic insect ecology. Ecology and classification of North American freshwater invertebrates.

[b29-tlsr-28-2-9] Jardine TD, McGreachy SA, Paton CM, Savoie M, Cunjak RA (2003). Stable isotopes in aquatic systems: Sample preparation, analysis and interpretation (Report No. 2656. Canadian Manuscript Report of Fisheries and Aquatic Sciences.

[b30-tlsr-28-2-9] Kominoski JS, Marczak LB, Richardson JS (2011). Riparian forest composition affects stream litter decomposition despite similarities in microbial and invertebrate comunities. Ecology.

[b31-tlsr-28-2-9] Lau DCP, Leung KMY, Dudgeon D (2008). Experimental dietary manipulations for determining the relative importance of allochthonous and autochthonous food sources in tropical streams. Freshwater Biology.

[b32-tlsr-28-2-9] Lau DCP, Leung KMY, Dudgeon D (2009). Are autochthonous foods more important than allochthonous resources to benthic consumers in tropical head–water streams?. Journal of the North American Benthological Society.

[b33-tlsr-28-2-9] Lester PJ, Mitchell SF, Scott D, Lyon GL (1995). Utilization of willow leaves, grass and periphyton by stream macroinvertebrates: A study using stable carbon isotopes. Archiv fur Hydrobiologie.

[b34-tlsr-28-2-9] Macko SA, Ostrom NE, Lajtha K, Michener RH (1994). Pollution studies using stable isotopes. Stable isotopes in ecology and environmental science.

[b35-tlsr-28-2-9] March JG, Pringle CM (2003). Food web structure and basal food resource utilization along a tropical island stream continuum, Puerto Rico. Biotropica.

[b36-tlsr-28-2-9] Minagawa M, Wada E (1984). Stepwise enrichment of δ^15^N along food chains: further evidence and the relation between δ^15^N and animal age. Geochimica et Cosmochimica Acta.

[b37-tlsr-28-2-9] Morse JC, Yang L, Tian L (1994). Aquatic insects of China useful for monitoring water quality.

[b38-tlsr-28-2-9] Motta RL, Uieda VS (2004). Diet and trophic groups of an aquatic insect community in a tropical stream. Brazilian Journal of Biology.

[b39-tlsr-28-2-9] Munoz-Gil J, Marin-Espinoza G, Andrade-Vigo J, Zavala R, Mata A (2013). Trophic position of the Neotropic Cormorant (*Phalacrocorax brasilianus*): Integrating diet and stable isotope analysis. Journal of Ornithology.

[b40-tlsr-28-2-9] Nelson SM, Lieberman DM (2002). The influence of flow and other environmental factors on benthic invertebrates in the Sacramento River, U.S.A. Hydrobiologia.

[b41-tlsr-28-2-9] Newell RIE, Marshall N, Sasekumar A, Chong VC (1995). Relative importance of benthic microalgae, phytoplankton and mangroves as sources of nutrition for panaeid prawns and other coastal invertebrates from Malaysia. Marine Biology.

[b42-tlsr-28-2-9] Peterson BJ (1999). Stable isotope as tracers of organic matter input and transfer in benthic food webs: A review. Acta Oecologia.

[b43-tlsr-28-2-9] Peterson BJ, Fry B (1987). Stable isotopes in ecosystem studies. Annual Review of Ecology, Evolution and Systematics.

[b44-tlsr-28-2-9] Polis GA (1991). Complex trophic interactions in deserts: An empirical critique of food-web theory. American Naturalist.

[b45-tlsr-28-2-9] Post DM (2002). Using stable isotopes to estimate trophic position: Models, methods and assumptions. Ecology.

[b46-tlsr-28-2-9] Quinn JM, Cooper AB, Stroud MJ, Burrell GP (1997). Shade effects on stream periphyton and invertebrates: An experiment in streamside channels. New Zealand Journal of Marine and Freshwater Research.

[b47-tlsr-28-2-9] Roger PA, Reynaud PA (1979). Ecology of blue-green algae in paddy fields. Nitrogen and Rice.

[b48-tlsr-28-2-9] Rounick JS, Winterbourn MJ (1986). Stable carbon isotopes and carbon flow in ecosystems. Bioscience.

[b49-tlsr-28-2-9] Salas M, Dudgeon D (2001). Stable–isotope determination of mayfly (Insecta: Ephemeroptera) food sources in three tropical Asian streams. Fundamental and Applied Limnology.

[b50-tlsr-28-2-9] Singh RN (1961). Role of blue-green algae in nitrogen economy of Indian agriculture.

[b51-tlsr-28-2-9] Suren AM, Biggs BJF, Duncan MJ, Bergey L, Lambert P (2003). Benthic community dynamics during summer low-flows in two rivers of contrasting enrichment 2. Invertebrates. New Zealand Journal of Marine and Freshwater Research.

[b52-tlsr-28-2-9] Thomas JD, Daldorph PW (1994). The influence of nutrient and organic enrichment on a community dominated by macrophytes and gastropod molluscs in a eutrophic drainage channel: Relevance to snail control and conservation. Journal of Applied Ecology.

[b53-tlsr-28-2-9] Thorp JH, Delong MD, Greenwood KS, Casper AF (1998). Isotopic analysis of three food web theories in constricted and floodplain regions of a large river. Oecologia.

[b54-tlsr-28-2-9] Torres-Ruiz M, Wehr JD, Perrone AA (2007). Trophic relationships in a stream food web: importance of fatty acids for macroinvertebrate consumers. Journal of the North American Benthological Society.

[b55-tlsr-28-2-9] Vander Zanden MJ, Cabana G, Rasmussen JB (1997). Comparing trophic position of freshwater fish calculated using stable nitrogen isotope ratios (δ^15^N) and literature dietary data. Canadian Journal of Fish and Aquatic Sciences.

[b56-tlsr-28-2-9] VanDongen J, Poczekaj K, Snyder E (2011). Understanding nutrient pathways and reciprocal subsidies between stream and riparian zones using stable isotope analysis.

[b57-tlsr-28-2-9] Vannote RL, Minshall GW, Cummins KW, Sedell JR, Cushing CE (1980). The river continuum concept. Canadian Journal of Fisheries and Aquatic Sciences.

[b58-tlsr-28-2-9] Venkataraman GS (1972). Algal biofertilizers and rice cultivation.

[b59-tlsr-28-2-9] Yule CM, Yong HS (2004). Freshwater invertebrates of the Malaysian region.

[b60-tlsr-28-2-9] Zulkifli SZ, Mohamat-Yusuff F, Ismail A, Miyazaki N (2012). Food preference of the giant mudskipper *Periophthalmodon schlosseri* (Teleostei: Gobiidae). Knowledge and Management of Aquatic Ecosystems.

[b61-tlsr-28-2-9] Zulkifli SZ, Mohamat-Yusuff F, Mukhtar A, Ismail A, Miyazaki N (2014). Determination of food web in intertidal mudflat of tropical mangrove ecosystem using stable isotope markers: A preliminary study. Life Science Journal.

